# Clinical and LAT1 Biomarker Correlates of Clinical Benefit from Nanvuranlat (JPH203) in Advanced Biliary Tract Cancer: A Post Hoc Analysis

**DOI:** 10.3390/cancers18152492

**Published:** 2026-08-04

**Authors:** Eric K. Rowinsky, Ghassan K. Abou-Alfa, Junji Furuse, Makoto Ueno, Masafumi Ikeda, Hiroko Tabuchi, Kazuo Sekiguchi, Michael Szarek

**Affiliations:** 1 Uniphar Development, LLC, Rockville, MD 20850, USA; 2 Memorial Sloan-Kettering Cancer Center, New York, NY 10065, USA; 3 Weill Cornell College, Cornell University, New York, NY 10065, USA; 4 School of Medicine, Trinity College Dublin, D02 PN40 Dublin, Ireland; 5 Kanagawa Cancer Center, Yokohama 241-8515, Japan; 6 National Cancer Center Hospital East, Kashiwa 277-8577, Japan; 7 ssg medical Inc., Kumamoto 860-0803, Japan; 8 J-Pharma Co., Ltd., Tokyo 105-0013, Japan; sekiguchi.k@j-pharma.com; 9 Division of Cardiology, University of Colorado Anschutz School of Medicine, Aurora, CO 80045, USA; 10 CPC Clinical Research, Aurora, CO 80045, USA; 11 School of Public Health, State University of New York, Downstate Health Sciences University, Brooklyn, NY 11203, USA; 12 Mount Sinai Fuster Heart Hospital, Icahn School of Medicine at Mount Sinai, New York, NY 10029, USA

**Keywords:** biliary tract cancer, LAT1, nanvuranlat, exploratory subgroup analysis, treatment-effect heterogeneity, translational oncology, cholangiocarcinoma

## Abstract

**Background/Objectives:** This exploratory post hoc analysis evaluated clinical and biomarker-defined treatment effects and accumulated nanvuranlat exposure in advanced biliary tract cancer (BTC). **Methods:** BICR-assessed progression-free survival (PFS) and overall survival (OS) were analyzed in the randomized Phase 2 full analysis set of 104 patients (nanvuranlat, *n* = 69; placebo, *n* = 35). Formal treatment-by-subgroup interaction tests assessed prior primary tumor resection status, LAT1 expression, and anatomical BTC subtype, evaluated both as a four-category variable and as pooled IHC/EHC/GBC versus AVC. Accumulated-exposure analyses pooled Phase 1 and Phase 2 data and were descriptive. **Results:** Median PFS was 46 versus 43 days (HR, 0.557; 95% CI, 0.344–0.903), and median OS was 155 versus 144 days (HR, 0.875; 95% CI, 0.551–1.390). Interaction tests were nominally significant for resection status with OS (*p* = 0.028) and for the four-category BTC-subtype variable with PFS (global *p* = 0.035), but not for LAT1 expression (PFS, *p* = 0.157; OS, *p* = 0.586) or pooled IHC/EHC/GBC versus AVC (PFS, *p* = 0.511; OS, *p* = 0.208). The binary and four-category subtype analyses addressed different hypotheses and were not considered contradictory. Higher accumulated-exposure quartiles showed numerically longer survival, but these analyses were susceptible to immortal-time bias, reverse causation, and time-dependent confounding. **Conclusions:** Nanvuranlat was associated with a lower hazard of progression or death than placebo, whereas the OS estimate was less conclusive. The subgroup and exposure findings remain exploratory but support prospective evaluation of resection status, anatomical subtype, and LAT1 expression.

## Simple Summary

Nanvuranlat is a selective inhibitor of L-type amino acid transporter 1 (LAT1) that is being developed for advanced biliary tract cancer. In this exploratory post hoc analysis of a randomized Phase 2 study, nanvuranlat was associated with a lower hazard of progression or death than placebo, whereas the overall-survival result was less conclusive. Formal interaction testing identified nominal signals for prior primary tumor resection status with overall survival and for the four-category anatomical BTC-subtype variable with progression-free survival; no statistically significant interaction was observed for LAT1 expression or for the pooled IHC/EHC/GBC-versus-AVC classification. The nonsignificant pooled binary interaction did not negate the four-category global signal because the analyses addressed different hypotheses. These analyses do not establish validated predictive factors; however, the randomized subgroup estimates, formal interaction signals, and biological rationale define clinically relevant hypotheses that merit focused prospective evaluation. Accumulated-exposure analyses were limited by their dependence on treatment duration and were interpreted as descriptive rather than causal.

## 1. Introduction

Biliary tract cancer (BTC) comprises a heterogeneous group of malignancies, including intrahepatic cholangiocarcinoma (IHC), extrahepatic cholangiocarcinoma (EHC), gallbladder cancer (GBC), and ampulla of Vater cancer (AVC). These anatomical subtypes differ in their epidemiology, etiology, molecular alterations, tumor microenvironment, clinical presentation, and treatment outcomes [1,2]. Large-scale international genomic and epigenomic studies have further demonstrated geographic and etiologic variation in the molecular landscape of cholangiocarcinoma, including distinct patterns in liver fluke-associated and non-fluke-associated tumors [2,3].

The treatment landscape for advanced BTC has evolved with the establishment of gemcitabine–cisplatin combined with durvalumab or pembrolizumab as first-line standards [4,5]. However, treatment options after progression on first-line therapy remain limited [6]. FOLFOX provides a modest overall-survival benefit compared with active symptom control, and liposomal irinotecan combined with fluorouracil and leucovorin has demonstrated activity in a randomized Phase 2b study, although the magnitude and regional reproducibility of the benefit have varied [7,8].

Cancer cells require an increased supply of essential nutrients to sustain proliferation and survival. L-type amino acid transporter 1 (LAT1), encoded by SLC7A5, mediates the cellular uptake of large neutral essential amino acids and is frequently overexpressed in BTC and other solid tumors [9]. High LAT1 expression has been associated with aggressive tumor characteristics and poor clinical outcomes in BTC [10,11,12]. LAT1 is also expressed by tumor-infiltrating immune cells, including CD4-positive T lymphocytes and macrophages, suggesting that LAT1-dependent amino acid metabolism may influence both cancer-cell growth and the tumor immune microenvironment [12,13,14,15]. Experimental studies further suggest that pharmacological inhibition of LAT1 can alter the immunosuppressive tumor microenvironment and enhance the antitumor activity of PD-1 blockade [15,16].

Nanvuranlat is a selective, non-transportable LAT1 inhibitor that suppresses essential amino acid uptake and downstream protein synthesis in cancer cells [17,18,19]. Preclinical studies have demonstrated antitumor activity in cholangiocarcinoma and other solid-tumor models [20,21]. A first-in-human Phase 1 study established a manageable safety profile and provided preliminary evidence of clinical activity in patients with advanced solid tumors, including BTC [22]. Subsequently, a randomized, double-blind, placebo-controlled Phase 2 study in patients with previously treated advanced BTC demonstrated an improvement in progression-free survival with nanvuranlat, whereas the overall-survival estimate was less conclusive [23].

The randomized design of the Phase 2 study provides an opportunity to explore whether treatment-effect estimates differ according to baseline clinical or biological characteristics. Anatomical BTC subtype, prior primary tumor resection status, and tumor LAT1 expression are clinically or biologically plausible factors for investigation. However, differences between subgroup-specific hazard ratios, or statistical significance within one subgroup but not another, do not by themselves establish a predictive biomarker or differential treatment effect; formal treatment-by-subgroup interaction testing is required [24].

Pharmacokinetic analyses may also provide information relevant to clinical development. In the present dataset, however, accumulated nanvuranlat exposure was calculated through treatment discontinuation and was therefore dependent on the duration of treatment. Analyses based on this measure are consequently susceptible to immortal-time bias, reverse causation, and time-dependent confounding and can only be interpreted descriptively.

Accordingly, we conducted post hoc exploratory analyses of the randomized Phase 2 full analysis set to characterize progression-free and overall survival according to prior primary tumor resection status, anatomical BTC subtype, and tumor LAT1 expression and to formally evaluate treatment-by-subgroup interactions. We additionally performed non-inferential descriptive analyses of accumulated nanvuranlat exposure using pooled Phase 1 and Phase 2 data. These analyses were intended to generate hypotheses for prospective evaluation and were not designed to establish validated predictive biomarkers, patient-enrichment criteria, or causal exposure–efficacy relationships. The resulting hypotheses are being evaluated prospectively in the ongoing global Phase 3 BEACON-BTC study, for which overall survival is the primary endpoint (ClinicalTrials.gov identifier: NCT07265674) [25].

## 2. Materials and Methods

### 2.1. Study Population and Data Sources

This post hoc exploratory analysis used data from two clinical studies of nanvuranlat (JPH203) conducted in Japan.

The first study was a Phase 1, open-label, dose-escalation trial (JPH203-SBECD-PI; UMIN000016546) that evaluated the safety, tolerability, pharmacokinetics, and preliminary antitumor activity of nanvuranlat in patients with advanced solid tumors. Only patients with biliary tract cancer (BTC) were included in the present analyses.

The second study was a multicenter, randomized, double-blind, placebo-controlled Phase 2 trial (JPH203-SBECD-P2; UMIN000034080) conducted in Japanese patients with advanced BTC who were refractory to, intolerant of, or ineligible for standard chemotherapy.

The principal efficacy, clinical-subgroup, and biomarker analyses were conducted using the Phase 2 full analysis set (FAS), which comprised 69 patients assigned to nanvuranlat and 35 assigned to placebo. The pooled descriptive exposure–outcome analyses included patients from both the Phase 1 and Phase 2 studies, as described below.

All analyses presented in this manuscript were post hoc, exploratory, and hypothesis-generating and were not prespecified in the original study protocols.

### 2.2. Study Design and Treatment

In the Phase 1 study, patients received intravenous nanvuranlat at dose levels ranging from 12 to 85 mg/m^2^. The study used a dose-escalation design and included single-dose and repeated-dose treatment periods.

In the Phase 2 study, patients were randomized in a 2:1 ratio to receive nanvuranlat 25 mg/m^2^ or placebo. Treatment was administered as a 90-min intravenous infusion once daily for five consecutive days, followed by a nine-day treatment-free interval in each 14-day cycle. Treatment was continued until disease progression, unacceptable toxicity, withdrawal of consent, or investigator decision.

Randomization in the Phase 2 study was stratified according to BTC subtype and prior primary tumor resection status. Detailed study procedures and the primary efficacy and safety results have been reported previously.

### 2.3. Analysis Populations and Clinical Variables

Analyses comparing nanvuranlat with placebo were restricted to the randomized Phase 2 FAS.

Exploratory subgroup analyses were performed according to the following baseline characteristics:

• BTC subtype: intrahepatic cholangiocarcinoma (IHC), extrahepatic cholangiocarcinoma (EHC), gallbladder cancer (GBC), or ampulla of Vater cancer (AVC);
• Prior primary tumor resection status: yes or no;
• Tumor LAT1 expression level: high or low;
• Sex;
• Age: <65 or ≥65 years;
• Eastern Cooperative Oncology Group performance status: 0 or 1;
• Treatment line: second line, third line, or fourth line or later.

An additional comparison of the pooled IHC/EHC/GBC population with the AVC population was performed post hoc. The combined IHC/EHC/GBC classification and analyses combining this classification with LAT1 expression were data-derived and were not prespecified in the original study protocol.

Previous systemic anticancer treatments, including prior exposure to immune checkpoint inhibitors, were identified from patient-level treatment histories and summarized according to randomized treatment group.

### 2.4. Clinical Endpoints and Tumor Assessment

Clinical endpoints evaluated in the present analyses included overall survival (OS), progression-free survival (PFS) assessed by blinded independent central review (BICR), and percentage change from baseline in the sum of target-lesion diameters.

OS was defined as the time from randomization to death from any cause. Patients who were alive at the end of follow-up or lost to follow-up were censored on the last date on which they were known to be alive.

BICR-assessed PFS was defined as the time from randomization to the earliest occurrence of radiographic disease progression according to Response Evaluation Criteria in Solid Tumors version 1.1, as determined by BICR, or death from any cause. Patients without a PFS event were censored at the last tumor assessment showing no documented progression; when non-study anticancer therapy was started, censoring occurred at the last nonprogressive assessment before the new therapy. Missing or incomplete baseline tumor assessments were censored at randomization. Death before the first scheduled assessment or before the next scheduled assessment was counted as an event, whereas death or progression after more than one missed scheduled assessment was censored at the last nonprogressive assessment. Missing tumor-assessment data were not imputed.

Radiographic tumor assessments were performed every six weeks (±3 days) from randomization until radiographic disease progression or death, whichever occurred first. RECIST evaluations were completed within seven days after imaging. The same imaging modality and acquisition conditions used at baseline were maintained throughout the study whenever possible.

In the original Phase 2 study, tumor assessments were performed independently by investigators and by a blinded independent central imaging laboratory. The present post hoc PFS analyses used the BICR assessments.

The principal censoring conventions are summarized above; detailed conventions followed the Phase 2 analysis rules.

Percentage change in tumor size was calculated from the BICR-assessed target-lesion measurements according to RECIST version 1.1.

### 2.5. LAT1 Immunohistochemical Assessment

For patients with available archival tumor tissue, LAT1 expression was assessed using formalin-fixed, paraffin-embedded specimens. Tissue sections of 3–4 μm thickness were deparaffinized and incubated overnight at 4 °C with a proprietary mouse monoclonal anti-human LAT1 antibody (Lot No. F4A-20150128-0; J-Pharma Co., Ltd., Tokyo, Japan).

LAT1 expression was evaluated microscopically using an immunoreactive scoring system calculated by multiplying the staining-intensity score (0–3) by the distribution score representing the proportion of LAT1-positive tumor cells (0–3). All slides were independently evaluated by two pathologists who were blinded to clinical information, and discrepancies were resolved by consensus.

Immunoreactive scores of 4–9 were classified as high LAT1 expression, whereas scores of 0–3 were classified as low LAT1 expression. Patients without an evaluable LAT1 result were excluded from analyses according to LAT1 expression but remained in the other applicable Phase 2 FAS analyses.

### 2.6. Exploratory Subgroup and Interaction Analyses

Subgroup-specific PFS and OS estimates were evaluated in the randomized Phase 2 FAS. Kaplan–Meier curves were generated for the principal analyses according to prior primary tumor resection status and LAT1 expression level. Subgroup-specific hazard ratios were summarized using forest plots.

Formal treatment-by-subgroup interaction analyses were performed to assess statistical evidence of treatment-effect heterogeneity according to prior primary tumor resection status, anatomical BTC subtype, LAT1 expression level, and the pooled IHC/EHC/GBC-versus-AVC classification. Anatomical BTC subtype was first evaluated as a four-category variable comprising IHC, EHC, GBC, and AVC. A separate post hoc binary interaction analysis compared the pooled IHC/EHC/GBC population with AVC because this classification represented a development-relevant population-enrichment hypothesis. The four-category and binary interaction analyses addressed distinct statistical hypotheses: the former tested for any heterogeneity across the four individual anatomical subtypes, whereas the latter tested whether the average treatment effect in the pooled IHC/EHC/GBC population differed from that in AVC.

• The pooled IHC/EHC/GBC classification was data-derived and was not prespecified in the original Phase 2 protocol. All interaction analyses were therefore considered exploratory and hypothesis-generating. Subgroup-specific hazard-ratio estimates were interpreted together with confidence intervals, formal interaction tests, and biological plausibility and were not considered to establish validated predictive biomarkers or patient-enrichment criteria.

### 2.7. Exposure Assessment and Descriptive Exposure–Outcome Analyses

Nanvuranlat pharmacokinetics were characterized using a population pharmacokinetic model developed from plasma concentration–time data obtained in the Phase 1 and Phase 2 studies.

Individual accumulated exposure was defined as the sum of the model-predicted areas under the concentration–time curve for all nanvuranlat doses administered from treatment initiation through treatment discontinuation.

The pooled exposure–outcome dataset comprised 98 patients, including five patients from the Phase 1 study and 93 patients from the Phase 2 study. Among these, 63 nanvuranlat-treated patients had accumulated-AUC estimates and were divided into four exposure quartiles comprising 16, 16, 16, and 15 patients, respectively. The remaining 35 patients received placebo and therefore had no accumulated nanvuranlat exposure; they were retained as a reference group but were not assigned to an exposure quartile.

Accumulated AUC was evaluated descriptively using quartile-based summaries and graphical analyses treating accumulated AUC as a continuous variable. Because accumulated AUC was calculated through treatment discontinuation and was intrinsically dependent on the duration of treatment, these analyses were considered non-inferential and susceptible to immortal-time bias, reverse causation, and time-dependent confounding. No causal exposure–efficacy conclusions were drawn from these analyses.

Patient accounting and the detailed exposure–outcome results are provided in the Supplementary Materials.

### 2.8. Nonclinical Biliary Distribution Study

To investigate the biliary disposition of nanvuranlat and its primary metabolite, NAc-JPH203, a pharmacokinetic study was conducted in a bile duct-cannulated male cynomolgus monkey with the slow NAT2 phenotype. Nanvuranlat was administered as a single intravenous dose of 0.6 mg/kg, and serial plasma and bile samples were collected for pharmacokinetic analyses.

All animal procedures were reviewed and approved by the Institutional Animal Care and Use Committee of the testing facility and were conducted in accordance with applicable institutional regulations and guidelines for animal experimentation. Surgical procedures were performed under anesthesia, with perioperative analgesia and postoperative veterinary monitoring.

### 2.9. Statistical Analysis

PFS and OS distributions were estimated using the Kaplan–Meier method. Median survival times and corresponding 95% confidence intervals were calculated for each treatment group. Median follow-up was estimated using the reverse Kaplan–Meier method. Numbers at risk are displayed below the principal Kaplan–Meier curves at prespecified time points.

For the overall Phase 2 FAS analyses, hazard ratios and 95% confidence intervals comparing nanvuranlat with placebo were estimated using Cox proportional hazards models stratified by BTC subtype and prior primary tumor resection status.

The Cox-model specification was adapted to each subgroup analysis so that a variable fixed within a subgroup was not simultaneously used as a stratification factor:

• Analyses within prior primary tumor resection subgroups were stratified by BTC subtype only;
• Analyses within individual BTC subtypes were stratified by prior primary tumor resection status only;
• Analyses within LAT1-expression, sex, age, ECOG performance-status, and treatment-line subgroups were stratified by both BTC subtype and prior primary tumor resection status;
• Analyses comparing the pooled IHC/EHC/GBC population with AVC were stratified by prior primary tumor resection status only.

Accordingly, certain subgroup-specific hazard-ratio estimates differ slightly from those reported in the primary Phase 2 publication [23].

Formal interaction models included terms for randomized treatment, the subgroup variable, and the treatment-by-subgroup interaction. The treatment-by-resection interaction model was stratified by anatomical BTC subtype. The treatment-by-BTC-subtype interaction model was stratified by prior primary tumor resection status. For the four-category BTC-subtype variable, the overall interaction *p*-value was obtained using a three-degree-of-freedom global Wald test. The treatment-by-LAT1 interaction model was stratified by BTC subtype and prior primary tumor resection status. The binary interaction model comparing pooled IHC/EHC/GBC with AVC was stratified by prior primary tumor resection status, and its *p*-value was based on a Wald test of the treatment-by-classification interaction coefficient. Tied event times in all Cox proportional hazards models were handled using the Breslow method.

The four-category BTC-subtype interaction and the pooled IHC/EHC/GBC-versus-AVC interaction were not considered interchangeable. A statistically significant global interaction could reflect heterogeneity among any of the four individual subtypes, whereas the binary interaction specifically evaluated the proposed pooled classification.

Hazard ratios, 95% confidence intervals, and nominal Wald *p*-values were reported for subgroup-specific estimates. Formal treatment-by-subgroup interaction *p*-values were prioritized over comparisons of statistical significance within individual subgroups.

All statistical tests were two-sided. No adjustment for multiplicity or false-discovery-rate procedure was applied. Accordingly, all subgroup analyses and nominal *p*-values should be interpreted as exploratory and nonconfirmatory.

Subgroup and interaction analyses were performed using R version 4.5.1. The original accumulated-exposure analyses were performed using R version 4.2.0 (R Foundation for Statistical Computing, Vienna, Austria).

## 3. Results

### 3.1. Patient Population and Overall Efficacy

The Phase 2 full analysis set (FAS) comprised 104 patients, including 69 patients assigned to nanvuranlat and 35 assigned to placebo. The population included 44 patients with intrahepatic cholangiocarcinoma (IHC), 21 with extrahepatic cholangiocarcinoma (EHC), 24 with gallbladder cancer (GBC), and 15 with ampulla of Vater cancer (AVC). Baseline demographic and clinical characteristics were generally comparable between the treatment groups (Table 1).

Prior immune checkpoint inhibitor exposure was identified in 9 of 104 patients (8.7%), including 4 of 69 patients in the nanvuranlat group and 5 of 35 patients in the placebo group.

Using the full dataset through 30 November 2022, the median follow-up for the Phase 2 FAS, estimated using the reverse Kaplan–Meier method, was 946 days (95% CI, 713–1406).

PFS events occurred in 68 of 69 patients assigned to nanvuranlat and all 35 patients assigned to placebo. Median BICR-assessed PFS was 46 days (95% CI, 44–47) with nanvuranlat and 43 days (95% CI, 43–46) with placebo. The stratified hazard ratio was 0.557 (95% CI, 0.344–0.903; *p* = 0.0176).

For OS, 63 deaths occurred among 69 patients in the nanvuranlat group and 33 deaths occurred among 35 patients in the placebo group. Median OS was 155 days (95% CI, 125–206) and 144 days (95% CI, 101–198), respectively. The stratified hazard ratio was 0.875 (95% CI, 0.551–1.390; *p* = 0.5708).

### 3.2. Overview of Exploratory Subgroup Analyses

**Figure 1:**
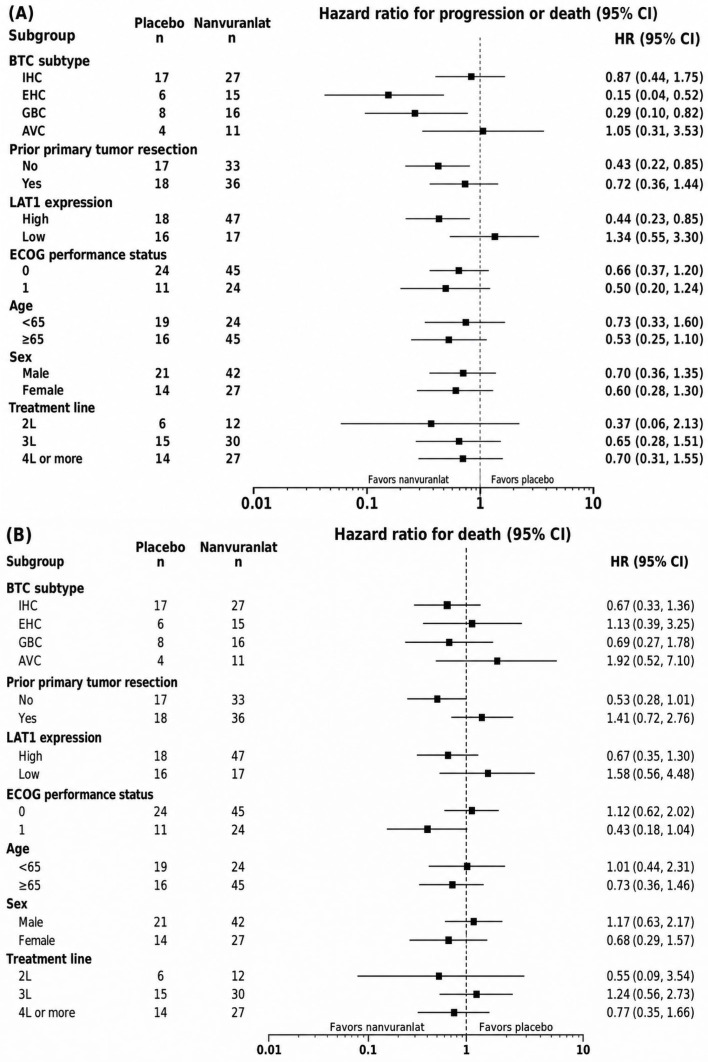
Forest plots of treatment-effect estimates across exploratory post hoc clinical and biomarker subgroups. Forest plots show hazard ratios (HRs) and 95% confidence intervals (CIs) for progression-free survival (**A**) and overall survival (**B**), comparing nanvuranlat with placebo in the Phase 2 full analysis set (FAS). Subgroups were defined according to BTC subtype, prior primary tumor resection status, LAT1 expression, ECOG performance status, age, sex, and treatment line. HRs were estimated using Cox proportional hazards models with stratification factors adapted to each analysis, as described in Section 2.9. The vertical dashed line indicates an HR of 1.0. Values to the left favor nanvuranlat, whereas values to the right favor placebo. All subgroup analyses were exploratory and were not adjusted for multiplicity; formal interaction tests are reported in the text.

Subgroup-specific treatment-effect estimates for PFS and OS in the Phase 2 FAS are summarized in Figure 1, and the formal treatment-by-subgroup interaction tests are summarized in Table 2. The analyses included BTC subtype, prior primary tumor resection status, LAT1 expression level, sex, age, ECOG performance status, and treatment line.

For PFS, hazard ratios were numerically below 1.0 in most subgroups, although the confidence intervals were generally wide because of the limited subgroup sample sizes. Lower PFS hazard ratios were observed in patients with EHC, GBC, no prior primary tumor resection, and high LAT1 expression.

For OS, the subgroup-specific estimates were less consistent and had wider confidence intervals. A numerically lower OS hazard ratio was observed among patients without prior primary tumor resection, whereas the estimates in several other subgroups were close to or above 1.0.

Formal treatment-by-subgroup interaction analyses are summarized in Table 2 and described in Section 3.3, Section 3.4 and Section 3.5. The four-category BTC-subtype global test and the pooled IHC/EHC/GBC-versus-AVC binary test addressed different hypotheses and were not treated as interchangeable. Because these analyses were post hoc and unadjusted for multiplicity, they are exploratory rather than confirmatory. Absence of nominal significance was not interpreted as proof of equivalent treatment effects.

### 3.3. Survival According to Prior Primary Tumor Resection Status

**Figure 2:**
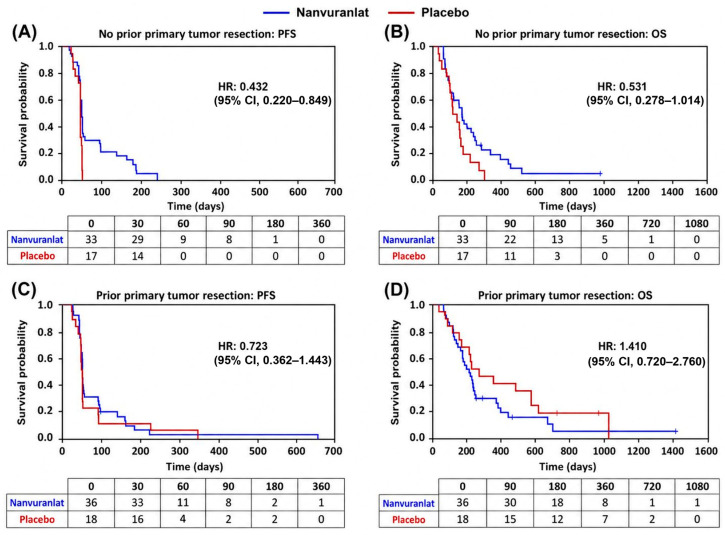
Kaplan–Meier curves according to prior primary tumor resection status. Kaplan–Meier curves for PFS and OS compare nanvuranlat with placebo within subgroups defined by prior primary tumor resection status. (**A**) PFS in patients without prior primary tumor resection. (**B**) OS in patients without prior primary tumor resection. (**C**) PFS in patients with prior primary tumor resection. (**D**) OS in patients with prior primary tumor resection.

Kaplan–Meier curves for PFS and OS according to prior primary tumor resection status are shown in Figure 2. Median follow-up was 946 days (95% CI, 259–946) in patients without prior primary tumor resection and 958 days (95% CI, 713–1406) in patients with prior primary tumor resection. PFS and OS analyses are summarized in Supplementary Table S1.

Among patients without prior primary tumor resection, PFS events occurred in all 33 patients assigned to nanvuranlat and all 17 patients assigned to placebo. Median PFS was 46 days (95% CI, 43–48) with nanvuranlat and 43 days (95% CI, 32–45) with placebo (Figure 2A). The hazard ratio, estimated using a Cox model stratified by BTC subtype, was 0.432 (95% CI, 0.220–0.849).

For OS in patients without prior primary tumor resection, 31 deaths occurred among 33 patients assigned to nanvuranlat, and all 17 patients assigned to placebo died. Median OS was 152 days (95% CI, 88–202) with nanvuranlat and 105 days (95% CI, 70–147) with placebo (Figure 2B). The corresponding hazard ratio was 0.531 (95% CI, 0.278–1.014).

Among patients with prior primary tumor resection, PFS events occurred in 35 of 36 patients assigned to nanvuranlat and all 18 patients assigned to placebo. Median PFS was 46 days (95% CI, 44–50) and 44 days (95% CI, 42–46), respectively (Figure 2C). The hazard ratio was 0.723 (95% CI, 0.362–1.443).

For OS in patients with prior primary tumor resection, 32 deaths occurred among 36 patients in the nanvuranlat group and 16 deaths occurred among 18 patients in the placebo group. Median OS was 191 days (95% CI, 125–216) with nanvuranlat and 200 days (95% CI, 130–562) with placebo (Figure 2D). The corresponding hazard ratio was 1.410 (95% CI, 0.720–2.760).

The treatment-by-resection interaction *p*-values were 0.123 for PFS and 0.028 for OS. Thus, the analysis did not provide statistically significant evidence of treatment-effect heterogeneity according to resection status for PFS, whereas nominal evidence of heterogeneity was observed for OS. The OS finding, together with directionally favorable PFS and OS estimates in patients without prior resection, prioritizes resection status for prospective evaluation; however, it remains exploratory because the analysis was post hoc, involved small subgroups, and was not adjusted for multiplicity.

Patients received nanvuranlat (blue solid line) or placebo (red solid line). Censored observations are indicated by plus symbols. The *x*-axis represents time, and the *y*-axis represents survival probability. Within prior-resection-status subgroups, HRs and 95% CIs were estimated using Cox proportional hazards models stratified by BTC subtype only. Numbers at risk are shown below each panel.

Preliminary nonclinical pharmacokinetic data from a cynomolgus monkey with the slow NAT2 phenotype demonstrated substantially higher concentrations of nanvuranlat and its major metabolite, NAc-JPH203, in bile than in plasma following a single intravenous administration of nanvuranlat (0.6 mg/kg, approximately equivalent to 25 mg/m^2^ in humans). For nanvuranlat, bile-to-plasma ratios exceeded 600-fold for Cmax and 8000-fold for AUC. Corresponding ratios for NAc-JPH203 exceeded 133,000-fold for Cmax and 947,000-fold for AUC (Supplementary Table S7). Although based on a single animal, these preliminary findings suggest preferential biliary distribution of nanvuranlat and warrant further investigation.

### 3.4. Treatment-Effect Estimates According to BTC Subtypes

Treatment-effect estimates differed numerically across the four anatomical BTC subtypes (Figure 1).

For PFS, the hazard ratios were 0.872 (95% CI, 0.436–1.746) in patients with IHC, 0.150 (95% CI, 0.044–0.515) in patients with EHC, 0.287 (95% CI, 0.101–0.818) in patients with GBC, and 1.048 (95% CI, 0.311–3.531) in patients with AVC.

For OS, the corresponding hazard ratios were 0.668 (95% CI, 0.327–1.363) for IHC, 1.131 (95% CI, 0.394–3.251) for EHC, 0.686 (95% CI, 0.265–1.780) for GBC, and 1.915 (95% CI, 0.516–7.103) for AVC. All subtype-specific OS confidence intervals included 1.0.

For the four-category anatomical BTC-subtype variable, the global treatment-by-subtype interaction *p*-value was 0.035 for PFS and 0.583 for OS. The nominal PFS interaction indicated potential heterogeneity across the four individual anatomical subtypes; however, the global test did not identify which subtype or subtypes accounted for the heterogeneity.

In the separate binary analysis, the hazard ratios in the pooled IHC/EHC/GBC population were 0.489 (95% CI, 0.288–0.831) for PFS and 0.764 (95% CI, 0.464–1.257) for OS, compared with 1.048 (95% CI, 0.311–3.531) and 1.915 (95% CI, 0.516–7.103), respectively, in AVC. The treatment-by-classification interaction *p*-values were 0.511 for PFS and 0.208 for OS. This analysis therefore did not provide statistical evidence that the average treatment effect in the pooled IHC/EHC/GBC population differed from that in AVC.

The four-category global and binary pooled-classification analyses addressed different hypotheses and should not be interpreted as contradictory. The binary analysis combined IHC, EHC, and GBC despite materially different subtype-specific hazard-ratio estimates and had limited power because the AVC subgroup included only 15 patients, including four placebo-treated patients. Notably, 13 of the 15 patients with AVC had undergone prior primary tumor resection, which may have also influenced the estimate in this small subgroup.

### 3.5. Exploratory Treatment-Effect Estimates According to LAT1 Expression

LAT1 expression status was evaluable in 98 of the 104 patients in the Phase 2 FAS. Sixty-five patients were classified as LAT1-high, including 47 assigned to nanvuranlat and 18 assigned to placebo. Thirty-three patients were classified as LAT1-low, including 17 assigned to nanvuranlat and 16 assigned to placebo. LAT1 expression status was unavailable for six patients.

**Figure 3:**
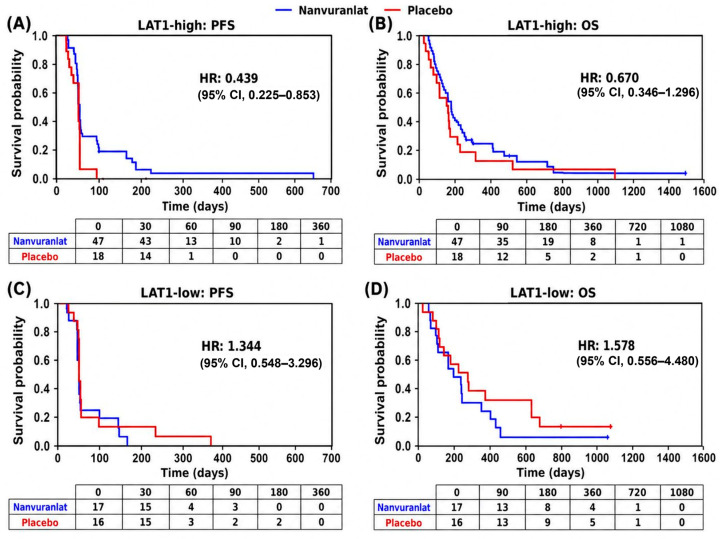
Progression-free survival and overall survival according to LAT1 expression level in the Phase 2 full analysis set. Kaplan–Meier curves compare nanvuranlat with placebo in patients with high or low LAT1 expression. Panel (**A**) shows BICR-assessed progression-free survival in the LAT1-high subgroup, panel (**B**) shows overall survival in the LAT1-high subgroup, panel (**C**) shows BICR-assessed progression-free survival in the LAT1-low subgroup, and panel (**D**) shows overall survival in the LAT1-low subgroup. Detailed Kaplan–Meier estimates are provided in Supplementary Table S2. Patients received nanvuranlat (blue solid line) or placebo (red solid line); censored observations are indicated by plus symbols, and numbers at risk are shown below each panel.

Kaplan–Meier curves for BICR-assessed PFS and OS according to LAT1 expression level are shown in Figure 3. Detailed Kaplan–Meier estimates are provided in Supplementary Table S2.

In the LAT1-high subgroup, PFS events occurred in 46 of 47 patients assigned to nanvuranlat and all 18 patients assigned to placebo. Median PFS was 47 days (95% CI, 46–49) with nanvuranlat and 43 days (95% CI, 32–46) with placebo (Figure 3A). The hazard ratio was 0.439 (95% CI, 0.225–0.853).

For OS in the LAT1-high subgroup, 42 deaths occurred among 47 patients assigned to nanvuranlat, and all 18 patients assigned to placebo died. Median OS was 153 days (95% CI, 110–191) and 130 days (95% CI, 68–147), respectively (Figure 3B). The hazard ratio was 0.670 (95% CI, 0.346–1.296). Median follow-up in the LAT1-high subgroup was 1406 days (95% CI, 272–1406).

In the LAT1-low subgroup, all 17 patients assigned to nanvuranlat and all 16 patients assigned to placebo experienced a PFS event. Median PFS was 43 days (95% CI, 40–46) with nanvuranlat and 43 days (95% CI, 42–47) with placebo (Figure 3C). The hazard ratio was 1.344 (95% CI, 0.548–3.296).

For OS in the LAT1-low subgroup, 16 deaths occurred among 17 patients assigned to nanvuranlat and 14 deaths occurred among 16 patients assigned to placebo. Median OS was 175 days (95% CI, 88–313) and 245 days (95% CI, 101–562), respectively (Figure 3D). The hazard ratio was 1.578 (95% CI, 0.556–4.480). Median follow-up in the LAT1-low subgroup was 946 days (95% CI, 713–958).

### 3.6. Descriptive Exposure–Outcome Analyses

The pooled exposure–outcome population comprised 98 patients from the Phase 1 and Phase 2 studies. Sixty-three nanvuranlat-treated patients had accumulated-AUC estimates available and were divided into four exposure quartiles comprising 16, 16, 16, and 15 patients, respectively. Thirty-five placebo-treated patients had no nanvuranlat exposure and were retained as the placebo reference group but were not assigned to an exposure quartile.

Descriptively, patients in the higher accumulated-exposure quartiles had longer observed PFS and OS and more favorable tumor-size changes than patients in the lower quartiles. However, accumulated AUC was calculated through treatment discontinuation and was therefore intrinsically dependent on treatment duration. These observations are consequently vulnerable to immortal-time bias, reverse causation, and time-dependent confounding and should not be interpreted as evidence of a causal exposure–efficacy relationship. Nevertheless, the directionally concordant findings at the exposure extremes across OS, PFS, and tumor-size change remain scientifically useful for generating a prospective regimen hypothesis.

Patient accounting, accumulated-AUC quartile distributions, Kaplan–Meier estimates, and tumor-size analyses are provided in Supplementary Figures S1–S3 and Supplementary Tables S3–S6.

## 4. Discussion

### 4.1. Overview of the Exploratory Findings

This post hoc analysis provides hypothesis-generating clinical and translational observations regarding nanvuranlat in advanced biliary tract cancer. In the randomized Phase 2 full analysis set, nanvuranlat was associated with a lower hazard of progression or death than placebo, whereas the overall-survival estimate was less conclusive. Exploratory analyses subsequently identified numerically different subgroup-specific treatment-effect estimates according to prior primary tumor resection status, anatomical BTC subtype, and tumor LAT1 expression.

Formal interaction testing provided two exploratory signals of treatment-effect heterogeneity: prior primary tumor resection status for OS and the four-category anatomical BTC-subtype variable for PFS. These findings were nominally significant but arose from post hoc analyses involving small subgroups and multiple correlated comparisons. They should therefore be regarded as focused hypotheses for prospective evaluation rather than confirmatory evidence.

No statistically significant interaction was observed for LAT1 expression or for the pooled IHC/EHC/GBC-versus-AVC classification. These nonsignificant results do not demonstrate equivalent treatment effects across the corresponding populations; rather, they indicate that the available dataset did not provide sufficient statistical evidence to confirm those specific interaction hypotheses. Taken together, the findings define a focused and biologically grounded framework for prospective testing but do not validate patient-enrichment criteria or predictive biomarkers.

### 4.2. Prior Primary Tumor Resection Status

Numerically lower hazard ratios for both PFS and OS were observed among patients without prior primary tumor resection than among those with prior resection. The treatment-by-resection interaction *p*-values were 0.123 for PFS and 0.028 for OS. The nominal OS interaction, coupled with directionally favorable PFS and OS estimates in unresected patients, elevates resection status beyond a purely descriptive subgroup observation and prioritizes it for prospective stratification. The signal remains nonconfirmatory because the analysis was post hoc, the subgroup sample sizes were small, multiple correlated analyses were performed, and no multiplicity adjustment was applied.

The biological basis for the observed difference according to resection status remains uncertain. Prior primary tumor resection may reflect differences in disease biology, tumor burden, metastatic pattern, timing of recurrence, or subsequent treatment history. It may also be associated with changes in biliary anatomy that could theoretically affect local drug distribution.

Preliminary nonclinical pharmacokinetic data from a bile duct-cannulated cynomolgus monkey demonstrated substantially higher concentrations of nanvuranlat and its metabolite in bile than in plasma. Although these observations raise the hypothesis that preservation of native biliary anatomy might influence local exposure, they were derived from a single animal and cannot explain the clinical subgroup findings. The biliary-distribution data should therefore be regarded as supportive biological context rather than mechanistic evidence.

Alternatively, prior primary tumor resection may simply be a surrogate for prognostic factors that were not fully measured or balanced in the present dataset. Accordingly, resection status should be considered a hypothesis-generating clinical variable requiring prospective evaluation rather than an established stratification or enrichment factor.

### 4.3. Anatomical BTC Subtype

The nominally significant four-category interaction for PFS suggests that the treatment-effect pattern may not be uniform across IHC, EHC, GBC, and AVC. This observation is biologically plausible because these anatomical subtypes differ in molecular alterations, cellular origin, metabolic dependencies, tumor microenvironment, disease course, and clinical management [1,2,3,26,27]. The global test, however, does not identify a single subtype responsible for the observed heterogeneity, and the subtype-specific estimates were imprecise because of the limited sample sizes.

The separate pooled IHC/EHC/GBC-versus-AVC interaction was not statistically significant for either PFS or OS. This result should not be interpreted as evidence that the treatment effects were equivalent in the pooled non-AVC population and AVC. The analysis had limited power because the AVC subgroup included only 15 patients, including only four placebo-treated patients. Moreover, pooling IHC, EHC, and GBC combined subtypes with materially different PFS hazard-ratio estimates and may have attenuated contrasts between individual subtypes.

Importantly, the nonsignificant binary interaction does not negate the nominal four-category global PFS interaction. The global test evaluated whether any heterogeneity was present across the four individual subtypes, whereas the binary test evaluated the narrower, data-derived hypothesis that IHC, EHC, and GBC could be treated as a single population distinct from AVC. The present results support continued prospective evaluation of anatomical subtype but do not validate the pooled IHC/EHC/GBC-versus-AVC classification as a treatment-selection rule.

### 4.4. LAT1 Expression as a Candidate Biomarker

Although formal treatment-by-LAT1 interaction testing was not statistically significant, the direction and magnitude of the subgroup-specific estimates remain biologically informative. LAT1-high tumors showed numerically lower hazard ratios than LAT1-low tumors for both PFS and OS, and LAT1 is the direct molecular target of nanvuranlat. The present study was not powered to detect a treatment-by-biomarker interaction in a subgroup analysis of 98 evaluable patients, particularly with only 33 patients in the LAT1-low population. Accordingly, the nonsignificant interaction should not be interpreted as evidence against LAT1 as a biomarker. Rather, LAT1 remains a mechanistically grounded candidate whose predictive and prognostic roles require prospective evaluation.

The present analysis cannot distinguish whether LAT1 expression is predictive of differential benefit from nanvuranlat, prognostic of clinical outcome irrespective of treatment, or both. High LAT1 expression has previously been associated with aggressive tumor biology and poor prognosis in BTC [10,11,12], and chance, limited power, wide confidence intervals, or baseline imbalances remain plausible alternative explanations for the observed effect-size differences.

The combined IHC/EHC/GBC and LAT1-high population was defined after examination of the Phase 2 data. It is therefore an exploratory, post hoc, data-derived subgroup and should not be interpreted as validation of a biomarker-enrichment strategy.

Accordingly, LAT1 expression should be regarded as a biologically plausible and clinically compelling candidate biomarker. Its predictive value is not established, but the present randomized effect-size pattern justifies prioritized prospective evaluation using standardized LAT1 assessment and adequately powered treatment-by-biomarker interaction analyses.

### 4.5. Interpretation of the Accumulated-Exposure Analyses

Exposure–response analyses can contribute to oncology drug development when exposure is defined independently of subsequent clinical outcomes [28,29,30]. In the present study, however, accumulated AUC was calculated from treatment initiation through treatment discontinuation and was therefore intrinsically dependent on the length of time that a patient remained alive and on treatment.

Numerically longer PFS and OS and more favorable tumor-size changes were observed in patients with higher accumulated exposure. These patterns are vulnerable to immortal-time bias, reverse causation, and time-dependent confounding [28,29,30]. In particular, patients must remain alive and continue treatment long enough to accumulate greater exposure. Longer survival can therefore produce a higher accumulated AUC, rather than a higher accumulated AUC necessarily producing longer survival.

The pooled exposure dataset also included patients from Phase 1 and Phase 2 studies that differed in dose, eligibility criteria, study design, and treatment setting. Although the patient-accounting table clarifies the populations included in the accumulated-AUC analysis, these sources of heterogeneity further limit interpretation.

Consequently, the accumulated-exposure findings cannot establish a causal exposure–efficacy relationship and should not independently support dose optimization. That limitation does not erase the observed pattern. Directionally concordant findings at the exposure extremes across OS, PFS, and tumor-size change are useful for generating regimen hypotheses. These data can identify which prospective questions to test; they cannot answer them. Alternative nanvuranlat regimens should therefore be compared prospectively using baseline randomization and concurrently collected efficacy, safety, tolerability, and pharmacokinetic data.

### 4.6. Relevance to Prospective Clinical Development

The hypotheses generated by the present analyses have been translated into falsifiable, prospectively defined evaluations in the ongoing global Phase 3 BEACON-BTC study. This represents an appropriate use of exploratory data to design a confirmatory test and should not be confused with retrospective validation of the subgroup or accumulated-exposure findings.

The ongoing global Phase 3 BEACON-BTC study is designed to evaluate nanvuranlat in a broader multinational population and in a treatment setting that more closely reflects contemporary management after platinum-based therapy. Alternative nanvuranlat regimens are evaluated prospectively through baseline randomization, and regimen selection is based on the totality of randomized efficacy, safety, tolerability, and pharmacokinetic data rather than on post-treatment accumulated-exposure classification.

BTC subtype is incorporated prospectively into the study design, and LAT1 expression and selected pharmacogenetic variables are assessed as exploratory factors. These analyses may help clarify whether the clinical and biomarker hypotheses generated by the Phase 1 and Phase 2 studies are reproducible. Definitive conclusions, however, require completion of the prospective study.

### 4.7. Limitations

Several limitations should be considered when interpreting these findings.

First, all subgroup analyses were post hoc and exploratory, involved multiple correlated comparisons, and were not adjusted for multiplicity. Several subgroups contained relatively few patients and events, resulting in wide confidence intervals and limited power for interaction testing. Nominally significant interaction *p*-values may therefore reflect chance and should not be regarded as confirmatory evidence.

Second, comprehensive genomic profiling was not prospectively required. Consequently, the present analysis could not determine whether LAT1 expression, resection status, or treatment outcomes were associated with molecularly defined subgroups, including FGFR2, IDH1, HER2, BRAF, NTRK, or mismatch-repair alterations. Anatomical subtype may also have served as an incomplete surrogate for underlying molecular heterogeneity.

Third, all participants were Japanese. Geographic and ethnic differences in BTC etiology, anatomical subtype distribution, molecular landscape, clinical management, and potentially nanvuranlat pharmacokinetics may limit generalizability to non-Japanese populations. Differences in the distribution of NAT2 acetylator phenotypes may be particularly relevant to nanvuranlat exposure. The findings require prospective validation in geographically and ethnically diverse populations.

Fourth, the Phase 2 study was conducted largely before chemoimmunotherapy became the standard first-line treatment for advanced BTC. Prior immune checkpoint inhibitor exposure was identified in only 9 of 104 patients in the Phase 2 FAS and was imbalanced between treatment groups, occurring in 4 of 69 patients assigned to nanvuranlat and 5 of 35 assigned to placebo. The potential influence of prior immunotherapy on nanvuranlat efficacy could therefore not be reliably evaluated, and the findings should not be directly extrapolated to patients treated after contemporary first-line chemoimmunotherapy.

Fifth, LAT1 analyses were limited to patients with evaluable archival tumor tissue. The tissue samples may have been collected at different stages of disease or before intervening therapies, and intratumoral or temporal heterogeneity in LAT1 expression could not be assessed. The study was also not sufficiently powered to distinguish the predictive and prognostic roles of LAT1.

Sixth, accumulated-exposure analyses were dependent on treatment duration and were subject to immortal-time bias, reverse causation, time-dependent confounding, and heterogeneity arising from pooling Phase 1 and Phase 2 data. These analyses should therefore be interpreted as descriptive only.

Finally, the hypothesis linking resection status, native biliary anatomy, and local drug exposure is based partly on preliminary pharmacokinetic findings from a single cynomolgus monkey. These nonclinical observations require independent confirmation and cannot establish a mechanism for the clinical subgroup findings.

These limitations define the evidentiary level rather than nullify the observed signals. The present analyses nominate prior primary tumor resection status, individual anatomical BTC subtype, and LAT1 expression for prioritized prospective testing, while not supporting the pooled IHC/EHC/GBC-versus-AVC classification as a validated treatment-selection rule. None should be used for treatment selection or patient enrichment before prospective randomized validation.

## 5. Conclusions

Formal interaction analyses identified nominal evidence of treatment-effect heterogeneity according to prior primary tumor resection status for OS and across the four anatomical BTC subtypes for PFS. Neither LAT1 expression nor the pooled IHC/EHC/GBC-versus-AVC classification showed a statistically significant interaction. However, the absence of statistical significance in these relatively small post hoc analyses does not establish equivalent treatment effects or exclude biologically meaningful subgroup differences. Taken together, the results define a focused and biologically coherent framework for prospective evaluation of resection status, individual anatomical BTC subtype, and LAT1 expression, while not validating the data-derived pooled IHC/EHC/GBC-versus-AVC classification as a treatment-selection rule.

Accumulated-exposure analyses were dependent on treatment duration and cannot support causal dose–response inference. Nevertheless, their directionally concordant descriptive patterns across OS, PFS, and tumor-size change support prospective randomized evaluation of alternative regimens. These prioritized hypotheses are being prospectively evaluated in contemporary BTC treatment settings in the ongoing multinational Phase 3 BEACON-BTC study (ClinicalTrials.gov identifier: NCT07265674).

## Figures and Tables

**Table 1:** Baseline demographic and clinical characteristics in the Phase 2 full analysis set (FAS).

Group	N	Treatment	Sex n	Age (years) n	Subtype n	ECOG Performance Status Score n	Treatment Line n	Primary Tumor Resection n	LAT1 Expression Level n
Placebo	35	-	Male: 21 Female: 14	<65: 19 ≥65: 16	IHC: 17 EHC: 6 GBC: 8 AVC: 4	0: 24 1: 11	Second-line: 6 Third-line: 15 Fourth-line or more: 14	Yes: 18 No: 17	High: 18 Low: 16 Unknown: 1
Nanvuranlat	69	25 mg/m^2^	Male: 42 Female: 27	<65: 24 ≥65: 45	IHC: 27 EHC: 15 GBC: 16 AVC: 11	0: 45 1: 24	Second-line: 12 Third-line: 30 Fourth-line or more: 27	Yes: 36 No: 33	High: 47 Low: 17 Unknown: 5

**Table 2:** Formal treatment-by-subgroup interaction analyses for progression-free survival and overall survival.

Subgroup Variable	Categories/Comparison	PFS Interaction *p*-Value	OS Interaction *p*-Value
Prior primary tumor resection	No versus yes	0.123	0.028
Anatomical BTC subtype	IHC, EHC, GBC, and AVC (global Wald test)	0.035	0.583
LAT1 expression	High versus low	0.157	0.586
Pooled anatomical classification	IHC/EHC/GBC versus AVC	0.511	0.208

Interaction *p*-values were obtained from Cox proportional hazards models containing randomized treatment, the subgroup variable, and the treatment-by-subgroup interaction. The four-category BTC-subtype interaction was evaluated using a three-degree-of-freedom global Wald test. The pooled IHC/EHC/GBC-versus-AVC analysis was a separate post hoc binary analysis and tested a different hypothesis from the four-category global test. Tied event times were handled using the Breslow method. All *p*-values are two-sided, nominal, and unadjusted for multiplicity.

## Data Availability

The datasets generated and/or analyzed during the current study are not publicly available due to confidentiality but are available from the corresponding author on reasonable request.

## Supplementary Materials

The following supporting information can be downloaded at: https://www.mdpi.com/article/10.3390/cancers18152492/s1: Table S1, Summary of the principal progression-free survival and overall survival analyses; Table S2, Kaplan–Meier estimates for progression-free survival and overall survival according to LAT1 expression level in the Phase 2 full analysis set; Table S3, Patient accounting for the pooled exposure–outcome population and accumulated-AUC quartile analysis; Table S4, Treatment exposure, clinical characteristics, and observed outcomes according to accumulated nanvuranlat exposure quartiles; Table S5, Baseline characteristics according to accumulated nanvuranlat exposure quartiles; Table S6, Descriptive overall-survival summaries according to accumulated nanvuranlat exposure quartiles; Table S7, Preliminary plasma and bile pharmacokinetic parameters of nanvuranlat and NAc-JPH203 in a cynomolgus monkey following a single intravenous dose of nanvuranlat (0.6 mg/kg) (Study No. JPH-P-2023-17); Figure S1, Descriptive overall survival according to accumulated nanvuranlat exposure quartiles. Kaplan–Meier curves show observed OS according to accumulated nanvuranlat exposure quartiles (Q1–Q4) and placebo. Patients treated with nanvuranlat were categorized into quartiles based on accumulated AUC calculated through treatment discontinuation. The Phase 2 placebo group was included as a descriptive reference. Because accumulated exposure depended on treatment duration, the curves are non-inferential and should not be interpreted causally.; Figure S2, Descriptive relationship between accumulated nanvuranlat exposure and overall survival. The scatter plot shows accumulated AUC and observed OS for individual patients; red circles indicate placebo-treated patients and blue circles indicate nanvuranlat-treated patients. Box plots summarize the observed OS distribution across placebo and exposure-quartile groups (Q1–Q4). The horizontal line represents the median, boxes indicate the interquartile range, and whiskers extend to the minimum and maximum values excluding outliers. This display is noninferential because accumulated exposure was calculated through treatment discontinuation and was therefore dependent on treatment duration.; Figure S3, Descriptive relationship between accumulated nanvuranlat exposure and change in tumor size. The scatter plot shows accumulated AUC and percentage change from baseline in the BICR-assessed sum of target-lesion diameters according to RECIST version 1.1. Each point represents an individual patient; red circles indicate placebo-treated patients and blue circles indicate nanvuranlat-treated patients. Negative values indicate tumor shrinkage and positive values indicate tumor growth. This display is exploratory and noninferential and should not be interpreted as demonstrating a causal exposure–response relationship.

## Abbreviations

The following abbreviations are used in this manuscript:

AUC	Area under the concentration–time curve
AVC	Ampulla of Vater cancer
BTC	Biliary tract cancer
BICR	Blinded independent central review
NAc-JPH203	N-acetylated metabolite of JPH203
CI	Confidence interval
Cmax	Peak drug concentration
ECOG	Eastern Cooperative Oncology Group
EHC	Extrahepatic cholangiocarcinoma
FAS	Full analysis set
GBC	Gallbladder cancer
HR	Hazard ratio
IHC	Intrahepatic cholangiocarcinoma
NAT2	N-acetyltransferase 2
LAT1	L-type amino acid transporter 1
OS	Overall survival
PFS	Progression-free survival
Q	Quartile
RECIST	Response Evaluation Criteria in Solid Tumors
Tmax	Time of peak drug concentration
