# Supplementary material for: Clinical and LAT1 Biomarker Correlates of Clinical Benefit from Nanvuranlat (JPH203) in Advanced Biliary Tract Cancer: A Post Hoc Analysis
Source: Cancers (Basel). 2026 Aug 4;18(15):2492. doi: 10.3390/cancers18152492 (PMC13465164; doi:10.3390/cancers18152492)
Supplement: Supplementary file 1 [file cancers-18-02492-s001.zip › cancers-4445531-supplementary.pdf]

Supplementary Table S1. Summary of the principal progression-free survival and overall survival analyses.

Analysis population	Endpoint	Treatment	N	Events, n	Median, days (95% CI)	HR (95% CI)
Phase 2 FAS	PFS	Nanvuranlat	69	68	46 (44–47)	0.56 (0.34–0.90)
		Placebo	35	35	43 (43–46)	Reference
	OS	Nanvuranlat	69	63	155 (125–206)	0.88 (0.55–1.39)
		Placebo	35	33	144 (101–198)	Reference
No prior resection	PFS	Nanvuranlat	33	33	46 (43–48)	0.43 (0.22–0.85)
		Placebo	17	17	43 (32–45)	Reference
	OS	Nanvuranlat	33	31	152 (88–202)	0.53 (0.28–1.01)
		Placebo	17	17	105 (70–147)	Reference
Prior resection	PFS	Nanvuranlat	36	35	46 (44–50)	0.72 (0.36–1.44)
		Placebo	18	18	44 (42–46)	Reference
	OS	Nanvuranlat	36	32	191 (125–216)	1.41 (0.72–2.76)
		Placebo	18	16	200 (130–562)	Reference

PFS was assessed by blinded independent central review according to RECIST version 1.1.

Supplementary Table S2. Kaplan–Meier estimates for progression-free survival and overall survival according to LAT1 expression level in the Phase 2 full analysis set.

LAT1 expression	Endpoint	Treatment	N	Events, n/N	Median, days (95% CI)	Median follow-up, days (95% CI)
High	PFS	Nanvuranlat	47	46/47	47 (46–49)	1406 (272–1406)
		Placebo	18	18/18	43 (32–46)	—
	OS	Nanvuranlat	47	42/47	153 (110–191)	—
		Placebo	18	18/18	130 (68–147)	—
Low	PFS	Nanvuranlat	17	17/17	43 (40–46)	946 (713–958)
		Placebo	16	16/16	43 (42–47)	—
	OS	Nanvuranlat	17	16/17	175 (88–313)	—
		Placebo	16	14/16	245 (101–562)	—

Median follow-up was estimated for the overall LAT1-high or LAT1-low subgroup and was not calculated separately by treatment group. PFS was assessed by blinded independent central review according to RECIST version 1.1.

Supplementary Table S3. Patient accounting for the pooled exposure–outcome population and accumulated-AUC quartile analysis.

Analysis stage	Study / treatment group	Total, n	Accumulated AUC available, n	Accumulated AUC unavailable, n	Accumulated AUC not applicable, n	Quartile assignment / analytical role
Source population	All patients	109	63	11	35	Includes Phase 1 and Phase 2 patients
	Phase 1 nanvuranlat-treated BTC patients	5	5	0	0	Included in pooled accumulated-AUC analysis
	Phase 2 full analysis set	104	58	11	35	Includes nanvuranlat and placebo groups
Phase 2 treatment group	Nanvuranlat	69	58	11	0	Patients with available AUC included in pooled quartile analysis
	Placebo	35	0	0	35	No nanvuranlat exposure; retained as descriptive reference group
Pooled exposure–outcome population	All evaluable patients	98	63	0	35	Includes 63 nanvuranlat-treated patients and 35 placebo-treated patients
Accumulated-AUC analysis	Nanvuranlat-treated patients with available accumulated AUC	63	63	0	0	Assigned to accumulated-AUC quartiles
Accumulated-AUC quartile	Quartile 1	16	16	0	0	Lowest accumulated-exposure quartile

Analysis stage	Study / treatment group	Total, n	Accumulated AUC available, n	Accumulated AUC unavailable, n	Accumulated AUC not applicable, n	Quartile assignment / analytical role
	Quartile 2	16	16	0	0	Second accumulated-exposure quartile
	Quartile 3	16	16	0	0	Third accumulated-exposure quartile
	Quartile 4	15	15	0	0	Highest accumulated-exposure quartile
Descriptive reference group	Placebo	35	0	0	35	Not assigned to an exposure quartile

The source population comprised five nanvuranlat-treated patients with BTC from the Phase 1 study and 104 patients from the Phase 2 full analysis set. Accumulated-AUC estimates were available for all five Phase 1 patients and 58 of 69 nanvuranlat-treated Phase 2 patients. Eleven nanvuranlat-treated Phase 2 patients without an accumulated-AUC estimate were excluded from quartile assignment. The pooled exposure–outcome population therefore comprised 63 nanvuranlat-treated patients with available accumulated-AUC estimates and 35 placebo-treated patients. Placebo-treated patients had no nanvuranlat exposure and were retained only as a descriptive reference group.

Supplementary Table S4. Treatment exposure, clinical characteristics, and observed outcomes according to accumulated nanvuranlat exposure quartiles.

Group	N	Dose	Phase	Primary tumor resection	BTC subtype	No. of doses median (min–max)	Observed OS (days), median (min–max)	Observed PFS (days), median (min–max)	% change in tumor diameter median (min–max)	Accumulated AUC (ng·h/mL) median (min–max)
Placebo	35	–	Phase 2: 35	Yes: 18 / No: 17	AVC: 4, IHC: 17, GBC: 8, EHC: 6	14 (4–166)	144.0 (22–1021)	43 (22–338)	20.30 (-7.3–53.9)	–
1st Quartile	16	25 mg/m²: 16	Phase 1: 1, Phase 2: 15	Yes: 7 / No: 9	AVC: 4, IHC: 5, GBC: 4, EHC: 3	9 (3–15)	71.5 (47–234)	41 (17–153)	22.65 (2.2–62.2)	5217 (2396–7227)
2nd Quartile	16	25 mg/m²: 16	Phase 2: 16	Yes: 9 / No: 7	AVC: 2, IHC: 7, GBC: 2, EHC: 5	15 (9–20)	171.5 (53–656)	45 (25–656)	18.8 (-5.6–52.3)	9426 (7997–10623)
3rd Quartile	16	25 mg/m²: 16	Phase 1: 1, Phase 2: 15	Yes: 9 / No: 7	AVC: 2, IHC: 8, GBC: 3, EHC: 3	15 (10–39)	178.5 (62–688)	46 (37–226)	18.15 (-18.9–49.6)	16226 (10989–20676)
4th Quartile	15	12 mg/m²: 1, 25 mg/m²: 12, 60 mg/m²: 2	Phase 1: 3, Phase 2: 12	Yes: 10 / No: 5	AVC: 3, IHC: 5, GBC: 4, EHC: 3	43 (8–215)	360.0 (84–1406)	130 (37–845)	1.60 (-57.2–65.9)	38980 (22088–174126)

OS and PFS medians in Table S4 are descriptive medians of the individual observed time-to-event values. Kaplan–Meier estimates of OS are reported separately in Table S6. PFS and tumor-size changes were based on BICR assessments according to RECIST version 1.1.

cancers

MDPI

Supplementary Table S5. Baseline characteristics according to accumulated nanvuranlat exposure quartiles.

Characteristic	Placebo (n=35)	Q1 (n=16)	Q2 (n=16)	Q3 (n=16)	Q4 (n=15)
**Age, median (range)**	63 (38-79)	66.5 (37-79)	71.5 (49-83)	68.5 (51-80)	72 (36-83)
**Age group, n (%)**					
**<65**	19 (54.3)	7 (43.8)	5 (31.3)	6 (37.5)	1 (6.7)
**≥65**	16 (45.7)	9 (56.3)	11 (68.8)	10 (62.5)	14 (93.3)
**Sex, n (%)**					
**Male**	21 (60.0)	8 (50.0)	14 (87.5)	11 (68.8)	8 (53.3)
**Female**	14 (40.0)	8 (50.0)	2 (12.5)	5 (31.3)	7 (46.7)
**ECOG performance status, n (%)**					
**0**	24 (68.6)	10 (62.5)	12 (75.0)	9 (56.3)	10 (66.7)
**1**	11 (31.4)	6 (37.5)	4 (25.0)	7 (43.8)	4 (26.7)*
**Treatment line, n (%)**					
**Second-line**	6 (17.1)	1 (6.3)	3 (18.8)	2 (12.5)	3 (20.0)
**Third-line**	15 (42.9)	7 (43.8)	8 (50.0)	7 (43.8)	3 (20.0)
**Fourth-line or more**	14 (40.0)	8 (50.0)	5 (31.2)	7 (43.8)	9 (60.0)

Baseline demographic and clinical characteristics of patients in the placebo group and nanvuranlat-treated patients stratified by quartiles of accumulated exposure (Q1–Q4), defined by accumulated AUC. Data are presented as median (range) for continuous variables and number (percentage) for categorical variables. * One subject in Q4 had missing ECOG performance status data at baseline.

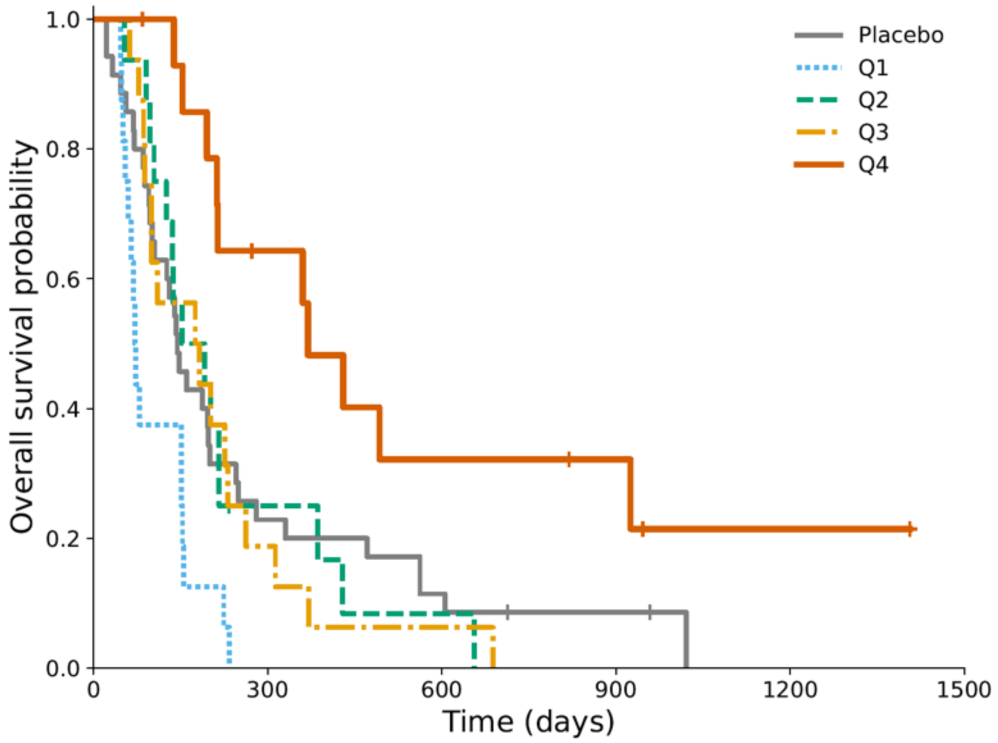

Supplementary Figure S1. Descriptive overall survival according to accumulated nanvuranlat exposure quartiles. Kaplan–Meier curves show observed OS according to accumulated nanvuranlat exposure quartiles (Q1–Q4) and placebo. Patients treated with nanvuranlat were categorized into quartiles based on accumulated AUC calculated through treatment discontinuation. The Phase 2 placebo group was included as a descriptive reference. Because accumulated exposure depended on treatment duration, the curves are noninferential and should not be interpreted causally.

Descriptively, median PFS ranged from 41 days in Q1 to 130 days in Q4. Because quartiles were defined using accumulated exposure through treatment discontinuation, this pattern is noninferential and may reflect immortal-time bias, reverse causation, and time-dependent confounding.

Supplementary Table S6. Descriptive overall-survival summaries according to accumulated nanvuranlat exposure quartiles.

Group	N	Kaplan–Meier median OS, days (95% CI)
Placebo	35	144.0 (105–245)
Quartile 1	16	71.5 (60–155)
Quartile 2	16	171.5 (125–NA)
Quartile 3	16	178.5 (99–313)
Quartile 4	15	369.0 (214–NA)

Kaplan–Meier median OS estimates are shown without inferential comparisons because accumulated AUC was dependent on treatment duration.

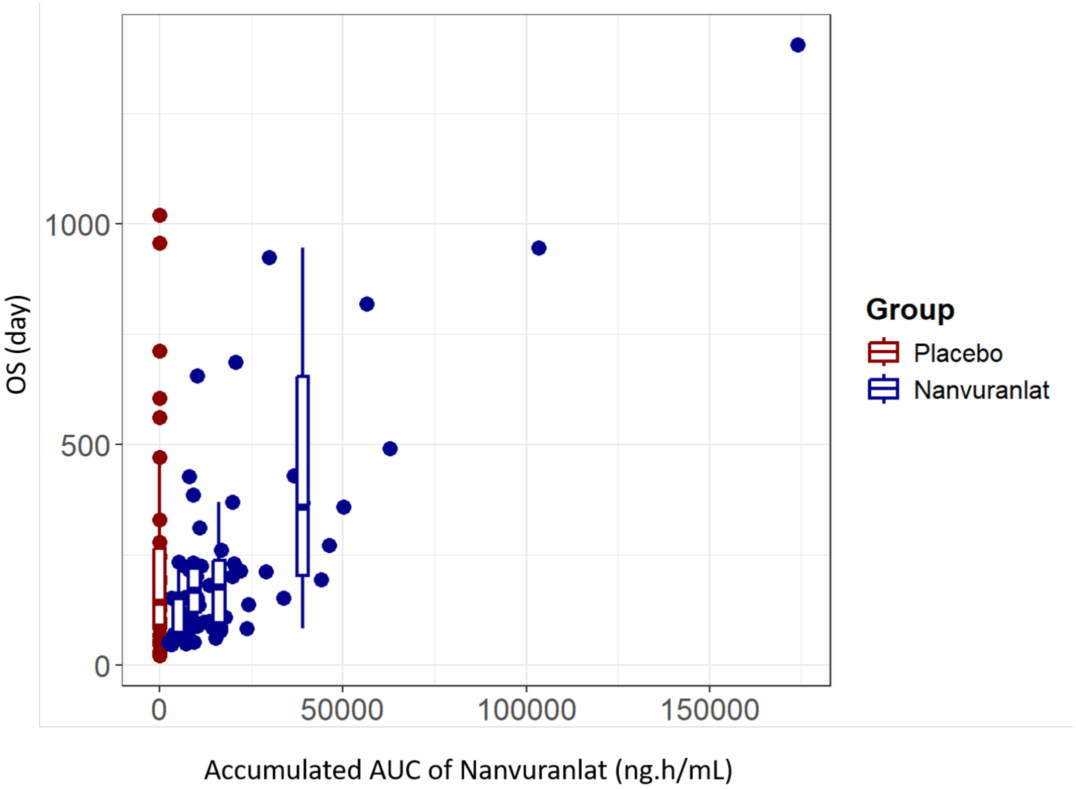

Supplementary Figure S2. Descriptive relationship between accumulated nanvuranlat exposure and overall survival. The scatter plot shows accumulated AUC and observed OS for individual patients; red circles indicate placebo-treated patients and blue circles indicate nanvuranlat-treated patients. Box plots summarize the observed OS distribution across placebo and exposure-quartile groups (Q1–Q4). The horizontal line represents the median, boxes indicate the interquartile range, and whiskers extend to the minimum and maximum values excluding outliers. This display is noninferential because accumulated exposure was calculated through treatment discontinuation and was therefore dependent on treatment duration.

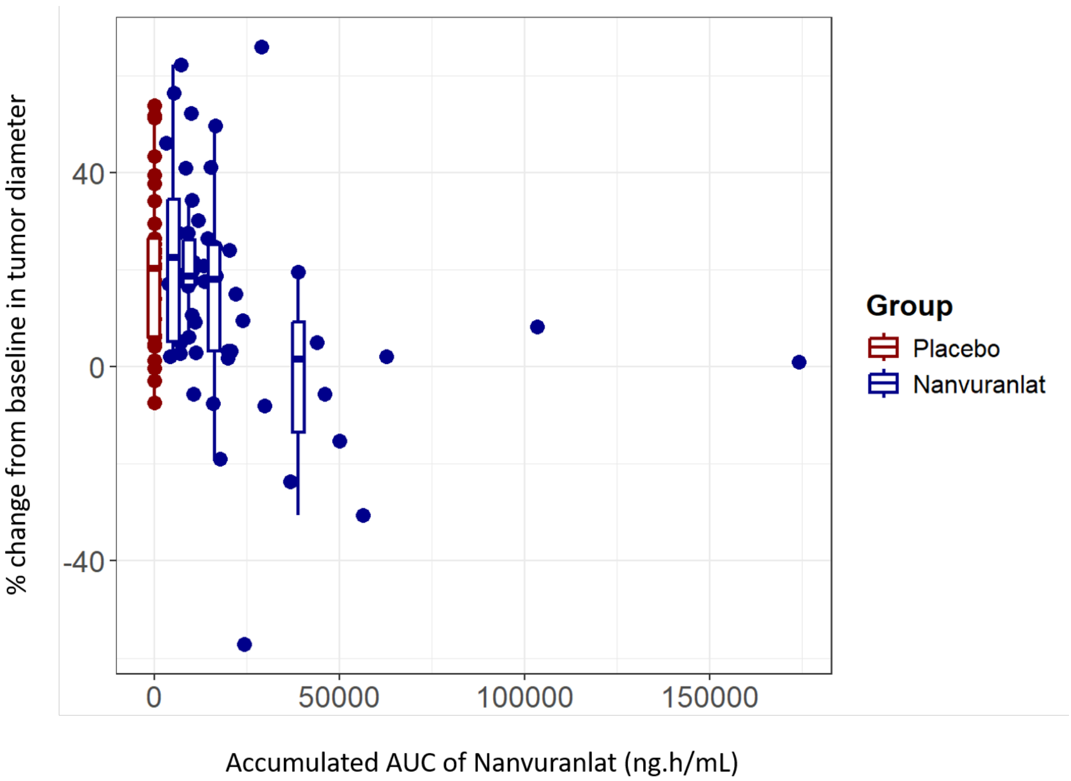

Supplementary Figure S3. Descriptive relationship between accumulated nanvuranlat exposure and change in tumor size. The scatter plot shows accumulated AUC and percentage change from baseline in the BICR-assessed sum of target-lesion diameters according to RECIST version 1.1. Each point represents an individual patient; red circles indicate placebo-treated patients and blue circles indicate nanvuranlat-treated patients. Negative values indicate tumor shrinkage and positive values indicate tumor growth. This display is exploratory and noninferential and should not be interpreted as demonstrating a causal exposure–response relationship.

Supplementary Table S7. Preliminary plasma and bile pharmacokinetic parameters of nanvuranlat and NAc-JPH203 in a cynomolgus monkey following a single intravenous dose of nanvuranlat (0.6 mg/kg) (Study No. JPH-P-2023-17).

Sample	Analyte	Cmax (ng/mL)	Tmax (h)	Bile: Plasma Cmax ratio	AUC (h·ng/mL)	Bile: Plasma AUC ratio
Plasma	Nanvuranlat	400.56	0	—	91.95	—
	NAc-JPH203	6.86	0.5	—	3.39	—
Bile	Nanvuranlat	269,000	2	672	815,074	8,864
	NAc-JPH203	913,000	2	133,090	3,210,984	947,193
